# Multisystem inflammatory syndrome in adults: a case report and review of the literature

**DOI:** 10.1186/s13256-022-03295-w

**Published:** 2022-03-03

**Authors:** Fardad Behzadi, Nicolas A. Ulloa, Mauricio Danckers

**Affiliations:** 1 Department of Internal Medicine, Aventura Hospital and Medical Center, Miami, FL USA; 2 Department of Emergency Medicine, Aventura Hospital and Medical Center, Miami, FL USA; 3 Department of Critical Care, Aventura Hospital and Medical Center, Miami, FL USA

**Keywords:** COVID, MIS-A, Organ failure, Pandemic, Case report

## Abstract

**Background:**

The current coronavirus disease pandemic has brought recognition of multisystem inflammatory syndrome in adults as a *de novo* entity, temporally associated with severe acute respiratory syndrome coronavirus 2 viral infection in adults. Hypothesis about its true pathophysiology remains controversial.

**Case report:**

The patient was a 22-year-old African American female presenting to the emergency department with fever, sore throat, and neck swelling for the past 3 days. During her initial emergency department visit, her blood pressure was stable at 110/57 mmHg, temperature of 39.4 °C, and heart rate of 150 beats per minute. While in the emergency department, she received broad-spectrum antibiotics (vancomycin and ceftriaxone) and 30 cc/kg bolus of normal saline. Originally, she was admitted to a telemetry floor. The following night, a rapid response code was called due to hypotension. At that time, her blood pressure was 80/57 mmHg. She appeared comfortable without signs of respiratory distress. She received intravenous fluids and vasopressors, and was transferred to the intensive care unit. The patient had reported a previous coronavirus disease infection a few weeks prior. She was diagnosed and treated for multisystem inflammatory syndrome in adults. Intravenous immunoglobulin infusion was initiated and completed on hospital day 5. She was weaned off vasopressors by day 6, and discharged home on day 11.

**Conclusion:**

Our case report is an example of the presentation, diagnosis, and management of multisystem inflammatory syndrome. Our research into previous case reports illustrates the wide range of presentations, degree of end organ damage, and treatment modalities. This diagnosis needs to be considered in the presence of recent coronavirus disease infection with new-onset end organ failure, as prompt diagnosis and treatment is crucial for better outcomes.

## Background

The current coronavirus disease (COVID-19) pandemic has brought the recognition of multisystem inflammatory syndrome in adults (MIS-A) as a *de novo* entity temporally associated with severe acute respiratory syndrome coronavirus 2 (SARS-CoV-2) viral infection in adults. Hypothesis about its true pathophysiology remains controversial. Its initial presentation, response to empiric therapy, and clinical outcomes are widely variable. We report the case of a 22-year-old female who presented with distributive shock after 3 days of fever, sore throat, and right-sided neck pain. She was diagnosed with MIS-A and successfully treated. We further provided the reader with an in-depth review of the current published case report of MIS-A available in the medical literature, and review the pathophysiology and clinical resemblance and difference to Kawasaki disease.

## Case description

A 22-year-old overweight African American female, with a body mass index (BMI) of 29.1 kg/m^2^, presented to the emergency department (ED) with 3 days of fever, sore throat, right-sided neck pain, and swelling. She denied any respiratory symptoms. She had tested positive for SARS-CoV-2 by polymerase chain reaction (PCR) 4 weeks prior, complaining of fever, chills, cough, headache, and diarrhea for 1 week. At that time, she had visited the ED and had been discharged with acetaminophen. Per the patient, she was not discharged with steroids or antibiotics.

During her initial ED visit, her blood pressure was stable at 110/57 mmHg, temperature of 39.4 °C, and heart rate of 150 beats per minute (BPM). While in the ED, she received broad spectrum antibiotics (vancomycin and ceftriaxone), 30 cc/kg bolus of normal saline, and blood cultures were obtained. Computed tomography (CT) of the neck with intravenous contrast revealed bilateral reactive lymphadenopathy with enlarged adenoids and mildly enlarged tonsillar pillars without abscesses. Initial chest X-ray was negative, without signs of pleural effusions or consolidations. Her electrocardiogram showed sinus tachycardia. She was admitted for persistent tachycardia and otolaryngology evaluation. Originally, the patient was admitted to a telemetry floor. The following night, a rapid response code was called due to hypotension. At that time, her blood pressure was 80/57 mmHg, heart rate was 125 BPM, respiratory rate of 25, and temperature of 103 F. She appeared comfortable, without signs of respiratory distress. She exhibited mild bilateral periorbital and lower extremities edema. Neck examination was notable for bilateral posterior lymphadenopathy with mild decreased range of motion. Her pulmonary and cardiac examinations were unremarkable other than tachycardia. Additionally, the rapid response team noted bilateral conjunctivitis as well as small strawberry rash diffusely. Another electrocardiogram was performed, which showed low voltage and sinus tachycardia. A point of care ultrasound (POCUS) was performed that was negative for pericardial effusion, right ventricular dilation, or signs of obstructive shock. She was fluid resuscitated with an additional 2 L of normal saline, with transient/negligible improvement of blood pressure. She was bolused another liter of lactated Ringer’s, initiated norepinephrine infusion, and admitted to the intensive care unit (ICU) for the management of distributive shock.

Her follow-up studies showed a peak d-dimer of 3557 ng/mL, C-reactive protein (CRP) of 47 mg/dL, and ferritin of 344 ng/mL. Fibrinogen was 460 mg/dL and remained within normal limits. She has a nadir hemoglobin of 10.6 g/dL, 24-hour urinary protein of 560 mg with preserved glomerular filtration rate through her entire hospital admission. Initial white blood cell count was 7000 cells/mm^3^ and only increased slightly after corticosteroid use. She exhibited a mild elevation of aspartate transaminase (AST) to 46 U/L, alanine transaminase (ALT) of 49 U/L, and alkaline phosphate (ALP) of 51 U/L. Her pro-B-type natriuretic peptide (BNP) was 3590 pg/mL on hospital day 2 and her troponin I peaked at 0.257 ng/m on day 3.

Official transthoracic echocardiography revealed a mild systolic dysfunction, grade 2 diastolic dysfunction and an ejection fraction of 40–45%, and a concentric small pericardial effusion. Coronary angiography revealed normal coronaries without evidence of obstruction or aneurysms. CT angiogram of the chest was negative for pulmonary embolism but notable for moderate-sized pleural effusions bilaterally. Cardiac magnetic resonance imaging (MRI) was not performed.

The patient received supportive treatment with dynamic hemodynamic-driven preload resuscitation and vasopressor support with norepinephrine. Her maximum dose of norepinephrine was 5 mcg/minute. Infectious disease was consulted on hospital day 3, who broadened antibiotic coverage with 3.375 mg piperacillin/tazobactam every 8 hours (q8) for 1 week. Broad infectious and immunologic workup was ordered and is summarized in Table 1. She tested negative for immunoglobulin (Ig)M and positive for IgG SARS-CoV-2 antibody. Dexamethasone 4 mg was initiated in the ED and continued q12 hours until hospital day 5 when it was changed by infectious disease team to hydrocortisone 50 mg q6 hours. Full-dose aspirin was initiated on hospital day 4 and continued until discharge. Intravenous immunoglobulin (IVIG) infusion was initiated and completed on hospital day 5, when she received 80 g over 16 hours. She was weaned off vasopressors by hospital day 6. An MRI of the neck without contrast on day 6 revealed resolution of her prevertebral soft tissue swelling and persistent nonspecific cervical lymphadenopathy bilaterally without any fluid collection. She received intravenous furosemide and albumin 25% intermittently with improvement in her interstitial edema. Blood and urine cultures remained negative during her hospitalization. She was discharged home on day 11.

**Table 1:** Infectious and immunologic panel

Test	Result	Test	Result
Hepatitis A IgM antibody	Negative	Human metapneumovirus (PCR)	Not detected
Hepatitis B surface antigen	Negative	Syphilis serology	< 0.2 AI
Hepatitis B core IgM antibody	Negative	Adenovirus (PCR)	Not detected
Hepatitis C antibody	Negative	*Bordetella holmesii* (PCR)	Not detected
HSV I IgG antibody	< 0.2 AI	*Bordetella pertussis* DNA (PCR)	Not detected
HSV II IgG antibody	< 0.2 AI	*Bordetella pertussis */bronchoscopy PCR	Not detected
HIV-1 and HIV-2 antigen and antibody	Nonreactive	Coxsackie type B (1) antibody	1:32 A
Influenza A (RT-PCR)	Not detected	Coxsackie type B (2) antibody	1:16 A
Influenza A H1 subtype (PCR)	Not detected	Coxsackie type B (3) antibody	1:16 A
Influenza A H3 subtype (PCR)	Not detected	Coxsackie type B (4) antibody	1:16 A
Influenza type B (PCR)	Not detected	Coxsackie type B (5) antibody	1:32 A
Parainfluenza 2 (PCR)	Not detected	Coxsackie type B (6) antibody	1:32 A
Parainfluenza 3 (PCR)	Not detected	CMV DNA (PCR)	Negative
Parainfluenza 4 (PCR)	Not detected	RSV type A (PCR)	Not detected
Group A strep screen	Negative	RSV type B (PCR)	Not detected
Anti-streptolysin O antibody	42 IU/mL	Rhinovirus (PCR)	Not detected
SARS-CoV-2 IgG antibody	Positive	EBV DNA	Positive
SARS-CoV-2 IgM antibody	Negative	Rheumatoid factor	Negative
IgG total	4247 mg/dL	ANA	Negative
IgG1	1545 mg/dL	C-ANCA	< 0.2 AI
IgG2	639 mg/dL	P-ANCA	< 0.2 AI
IgG3	110 mg/dL	dsDNA antibody	< 1 IU/mL
IgG4	44 mg/dL	Complement C3	70 (L) mg/dL
IgA	63.6 mg/dL	Complement C4	< 8 (L) mg/dL

*RT-PCR* reverse transcription-polymerase chain reaction, *HSV* herpes simplex virus, *HIV* human immunodeficiency virus, *CMV* cytomegalovirus, *RSV* respiratory syncytial virus, *EBV* Epstein–Barr virus, *dsDNA* double strain DNA antibodies, *ANA* antinuclear antibody, *C-ANCA* antineutrophil cytoplasmic antibodies, *P-ANCA* perinuclear anti-neutrophil cytoplasmic antibodies, *IgM* Immunoglobulin M, *IgG* Immunoglobulin G, *IgA* Immunoglobulin A

## Discussion

Multisystem inflammatory syndrome in adults (MIS-A) was first mentioned in 2020 following the initial description of this syndrome in the pediatric population (multi-inflammatory syndrome in children) during the COVID-19 pandemic. Since its first recognition, several case reports have been published in the literature, with a wide range of clinical manifestations and therapeutic interventions. MIS-A is suspected to be caused by an abnormal immune response to SARS-CoV-2 infection and is commonly associated with clinical features such as fever, systemic inflammation, and shock with end-organ damage [1, 2]. Many of these features have been proposed to resemble Kawasaki-like manifestations [1, 2]. According to the Centers of Disease Control (CDC), five criteria should be fulfilled to diagnosed MIS-A: (1) concurrent or previous (within the past 12 weeks) COVID-19 diagnosed by either PCR or antigen/antibody testing, (2) severe sickness necessitating hospitalization in those aged 21 years or more, (3) marked involvement or dysfunction of single or multiple extrapulmonary organs (acute kidney injury, acute liver injury, neurological involvement, cardiac insult, shock, hypotension, and so on), (4) absence of severe respiratory affection (respiratory signs and symptoms), and (5) exhibiting severe inflammation as per laboratory findings: elevated CRP, d-dimer, serum ferritin, erythrocyte sedimentation rate (ESR), fibrinogen, interleukin-6 (IL-6) [3]. In our case, the patient fulfilled all five criteria to make the diagnosis.

Thirty-six documented cases of MIS-A were reviewed and are summarized in Table 2. The mean age of patients was 33 years, with male predominance (23/36; 63%). Most of the patients had no past medical history of significance (23/36; 63%), while 17/36 (47%) contracted SARS-CoV-2 infection, suggested by PCR, antibody testing, or clinically. Fever was recorded in 31/36 cases (86%). Gastrointestinal symptoms were less frequently reported: nausea (7/36, 19%), abdominal pain (11/36; 30%), vomiting (5/36; 13%), and diarrhea (7/36; 19%). Like our case report, sore throat was present in five patients (5/36; 14%) [4–8] and unilateral cervical pain/swelling in four other cases (6/36; 16%) [8–12]. Some patients had predominant visual symptoms [5, 13–17].

**Table 2:** MIS-A published case reports

Authors	Age, sex, ethnicity	Past medical history	Signs and symptoms at presentation	Previous COVID-19 infection	Initial COVID-19 testing	ICU stay	Laboratory findings	Imaging studies	Treatments	Outcome
Kofman, 2020 [4]	25, female	None	Fever, dyspnea, sore throat, diarrhea, vomiting, cough, and adenopathy	No	PCR (+) IgG (+)	Yes	Increased neutrophils, ESR, CRP, d-dimer, ferritin, Tn, and creatinine; lymphopenia	Chest X-ray and CT: No detected abnormalities CT angiography: dilated main pulmonary artery CT abdomen/pelvis: acute uncomplicated pancreatitis Echo: dilated IVC then right ventricular dysfunction	Aspirin, IVIG	Recovery
Fox, 2020 [9]	31, female, African-American	HTN, DM, and obesity (BMI 36.1 kg/m^2^)	Fever, tachycardia, left-sided neck pain, nausea, vomiting, and parotitis by examination	Yes, 12 days prior	PCR (−)	NR	Elevated d-dimer, lactic acid, CRP, and creatinine	CT neck: bilateral parotid enlargement and swelling of the posterior nasopharynx to the oropharynx CT chest: bilateral basal GGO plus anterior mediastinal lymphadenopathy	NR	Deceased
Shaigany, 2020 [8]	45, male, Hispanic	No PMH BMI of 26.6 kg/m^2^	Fever, diarrhea, sore throat, painful lower extremities, diffuse exanthema, conjunctivitis, periorbital edema, left neck swelling with lymphadenopathy, plaques and papules diffuse, hypotension, tachycardia, and atrial fibrillation	No	PCR (+)	No	Increased neutrophils, low lymphopenia, ESR, CRP, d-dimer, ferritin, Tn, AST, ALT, PCT (3179 ng/mL), IL-6 (117 pg/mL)	Chest X-ray: diffuse interstitial haziness CT neck with contrast: inflamed edematous lower eyelids and preseptal spaces, reactive lymphadenopathy ECG: anterolateral ST segment elevation PCI: normal coronary TTE: global hypokinesia of the left ventricle with reduced EF of 40 Slit-lamp examination: conjunctivitis and uveitis	Full dose enoxaparin, IVIG (2 g/kg over 2 days), and single dose of IL-6 inhibitor (tocilizumab)	Recovery
Ahsan, 2020 [13]	28, male	Thalassemia minor. BMI of 28.48 kg/m^2^	High-grade fever (40.6 °C), anorexia, vomiting, nausea, lower limb pain, generalized weakness, red eye, difficult urination, and constipation. Bilateral facial nerve palsy, optic neuritis	Yes, 2 weeks before Ab (+), PCR (−)	Not done	NR	Anemia hypoalbuminemia leukocytosis with neutrophilia Elevated ESR, ferritin, and CRP	ECG: normal Chest X-ray: normal MRI brain and orbit: normal	Ceftriaxone 2 g daily and prednisolone 1 mg/kg/day orally for 6 weeks	Recovery
Bettach, 2021 [14]	54, female	None	Fever, septic shock, GI symptoms, skin rash, heart failure, bilateral acute anterior uveitis	No	PCR (−) IgG (+)	Yes	NR	Slit-lamp examination: bilateral corneal edema with Descemet’s membrane and keratin precipitates Fundus examination: small localized intracranial bleed Fluorescein angiography: no vascular abnormalities	Antibiotics, corticosteroids, and vasopressors. After 2 weeks, topical dexamethasone	Recovery
Razavi, 2020 [15]	23, male, African-American	BMI of 35.4 kg/m^2^	Fever, fatigue, myalgia, dyspnea, orthopnea, watery diarrhea, and temporal headache. Hypotension, bilateral scleral, and conjunctival injection	Yes, 1 month prior	PCR (−) IgG (+)	NR	Leukocytosis, lymphocytopenia, high Tn I and BNP (NSTEMI) High CRP, d-dimer, ferritin, and fibrinogen	Echo: global hypokinesia with reduced EF (40–45%) Chest X-ray: no focal consolidations CT chest with contrast: no abnormalities Cardiac MRI: pericardial effusion and borderline EF (54%)	Antibiotics, IVIG, methylprednisolone, aspirin, enoxaparin	Recovery
Gulersen, 2021 [18]	31, female	Obesity, asthma, pregnant (28 weeks)	Fever, left-sided pleuritic chest pain, shortness of breath. Late-onset hypotension and tachypnea	Yes, 4 weeks prior. PCR (+)	PCR (−) IgG (+)	Yes	Leukocytosis. Elevated CRP, normal lactate, ferritin, PCT, late-onset increased in cardiac enzymes and inflammatory markers	CT angiography of the chest: normal with no pulmonary embolism or lung pathology detected TTE: On admission, EF 65–70% with a hyperdynamic left side, rim pericardial effusion, and well-functioning right ventricle. On day 4: global dysfunction of the right and left ventricles with rim pericardial effusion Non-stress test: reactive fetus	Intravenous heparin, IVIG, dexamethasone (10 mg every 6 hours), mechanical ventilation, inotrope and vasopressor	Extubated on day 8, elective delivery, and discharged home on day 15
Malangu, 2020 [19]	46, male	History of pneumonia	Fever (39.1 °C), atrial fibrillation, mild hypoxia (SatO_2_ 91% on room air), bilateral exudative conjunctival injection, oral mucositis, bilateral cervical lymphadenopathy, and macular skin rash	No	PCR (−) IgG (+)	NR	Leukocytosis and thrombocytopenia. Elevated d-dimer, CRP, ferritin, LDH fibrinogen. Mildly elevated ALT, AST, kidney injury with hematuria, and proteinuria	CT angiography of the chest: bilateral apical patchy consolidations Chest X-ray: basal and middle lobe opacities TTE: left ventricular dysfunction with EF 31% and eccentric hypertrophy Cardiac MRI: perihilar lymph nodes with no infiltrative lesions Bronchoscopy: no malignant cells	Antibiotics and apixaban	Recovery
Othenin-Girard, 2020 [20]	22, male, East African	None	Five days of chills, myalgia, asthenia, diarrhea, and abdominal pain. Three weeks of loss of taste and smell sensations, and 1 day of dry cough, odynophagia, and rash (over trunk, extremities, palms)	Yes, 3 weeks prior. IgG (+)	PCR (+) IgG (+)	Yes	Leukocytosis, elevated CRP (275 mg/L), fibrinogen (8.5 g/L), d-dimer (3322 ng/mL), and creatinine (1.5 mg/dL) Autoimmune workup: negative ANA, ANCA, and rheumatoid factor	CT abdomen and chest: normal lung parenchyma with pulmonary embolism and inflamed mesenteric lymph nodes TTE: biventricular dysfunction/endomyocardial biopsy: myocarditis with necrotic foci Nerve conduction study: mononeuritis multiplex	IVIG, tocilizumab, rituximab, corticosteroids, and cyclophosphamide. Mechanical ventilation and extracorporeal membrane oxygenation (ECMO)	Recovery
Moghadam, 2020 [16]	21, male, Caucasian	None	Seven days of fever (40 °C), watery non-bloody diarrhea, chest tightness, vasoplegic shock, rash, tachypnea, bilateral conjunctivitis, and truncal and palmar rash	No	PCR (−) IgG (+)	Yes	Leukocytosis, CRP (365 mg/L), PCT (3.4 ng/mL), ferritin (1.282 mg/L), high lactate, Tn (55n ng/L)	Skin biopsy: inflammatory infiltrates TTE: hyperkinetic left ventricle with preserved EF CT scan chest and abdomen: compatible with congestive heart failure	Fluid resuscitation, noradrenaline, antibiotics (amikacin and ceftriaxone)	Recovery
Lidder, 2020 [5]	45, male	None	Five days of fever, red eyes, diarrhea, sore throat, eyelids edematous rash, nonexudative conjunctivitis, and abnormal perioral mucosa	No	PCR (+)	NR	Lymphopenia, elevated CRP, ESR, ferritin, d-dimer, and elevated Tn	TTE: global hypokinesia with reduced EF (40%) CT neck: unilateral lymphadenopathy	Eye-lubricating medications, topical prednisolone acetate 1%, IVIG, tocilizumab, and triamcinolone ointment for the rash	Recovery
Tung-Chen, 2021, Spain [6]	25, male	None	One-day history of nausea and abdominal pain. One week of fever (38 °C), sore throat, fatigue, anosmia, and orthopnea. Shock at presentation	No	PCR (−) IgM (+) IgG (+)	Yes	Lymphopenia (0.43 × 109/L), elevated fibrinogen (> 1200 mg/dL), CRP (337.1 mg/L), TnT I, and BNP	TTE: global hypokinesia with severely impaired left ventricular function (EF 29.7%) and rim pericardial effusion. EF improved after 8 days CT chest: no abnormalities Chest X-ray: no abnormalities ECG: sinus tachycardia with no other abnormalities	Antibiotics, ganciclovir, norepinephrine, milrinone, and diuretics	Recovery
Uwaydah, 2021 [7]	22, male	None	Four days of fever (39 °C), sore throat, diarrhea, nausea, vomiting, myalgia, headache, fatigue, erythematous rash involving the torso, tachycardia, hypotension, edema, and proteinuria	Yes, 40 days prior PCR (+)	PCR (−) IgG (+)	Yes	Leukocytosis, elevated creatinine, AST (53 U/L), ALT (81 U/L), direct bilirubin, CRP (249 mg/L), ferritin (4357 ng/mL), d-dimer (14 mg/mL), PCT (9 ng/mL), IL-6 (90 pg/mL), low platelets (122) and albumin (16 g/L)	TTE: severe tricuspid regurgitation, pulmonary HTN (46 mmHg), left ventricle dysfunction (EF 45%), and rim pericardial effusion. Normal echo after recovery CT chest: bilateral moderate pleural effusion and basilar atelectasis	Antibiotics, intravenous hydrocortisone	Recovery
Ahmad, 2021 [21]	26, male, Caucasian	None	Fever, abdominal pain, loose stool, nausea, reduced urine output, hypotension tachypnea (38 breath/minute) and hand/feet rash	PCR (+)	PCR (+) Abs (+)	Yes	Leukocytosis. Elevated lactic acid (9.7 mg/dL), CRP (246 mg/L), PCT (105.12 ng/mL), d-dimer (2.03), LDH (236 U/L), creatinine (4.66 mg/dL), and urea (38 mg/dL)	Lower limb doppler: left peroneal DVT Chest X-ray: peribronchial thickening Noncontrast CT abdomen: perinephric edema and mesenteric lymphadenopathy TTE: severely impaired left ventricular function (EF 15–20%) as well as right ventricular dysfunction. EF increased to 60% after 10 days	Vasopressors, IVIG, methylprednisolone (250 mg/6 hours), aspirin, anakinra (IL-1 receptor antagonist), mechanical ventilation, and CRRT	Recovery
Li, 2021 [10]	28, male	None	Five days of right-sided neck pain and swelling, enlarged tonsils, tenderness of the right submandibular fever, malaise, tachycardia, pruritic rash	4 weeks prior, PCR (+)	PCR (−) IgG (+)	NR	Leukocytosis (13,800/mm^3^), anemia (10.7 g/dL). Elevated hs-Tn I (11,908 ng/L), BNP (1661 pg/mL), CRP (304.2 mg/L), and ferritin (1588 mg/L)	CT neck: cervical lymphadenopathy, more on the right side TTE: mildly impaired left ventricular function (EF 45–55%) Cardiac MRI: rim pericardial effusion and slightly impaired right ventricular function	Broad-spectrum antibiotics, fluid resuscitation, beta-blocker, ACE inhibitor	Recovery
Veyseh, 2021 [23]	43, female	None	Fever, hypotension, tachycardia, erythematous rash, diarrhea, and cramping abdominal pain	No	PCR (−)	Yes	High WBCs, CRP, ferritin, d-dimer, fibrinogen, LDH, AST, and ALT	TTE: reduced EF (toxic cardiomyopathy), EF improved after IVIG and steroids	Antibiotics, vasopressors, IVIG, and intravenous solumedrol	Recovery
Diakite, 2021, [17]	33, male	HTN	Fever, diarrhea, chest pain, dyspnea, conjunctivitis, and cheilitis. Hypotension, tachycardia, and elevated hepatojugular reflux	Possible 6 weeks prior	PCR (−) IgG (+)	NR	Leukocytosis (21,000/mm^3^), anemia (10.7 g/dL), high AST, ALT, creatinine, CRP, d-dimer, BNP, and Tn	TTE: global hypokinesia, reduced EF (20%), and dilated IVC. Cardiac MRI revealed improved cardiac function after a week of treatment Coronary CT: aneurysms involving the right coronary, interventricular artery, and the left circumflex	Dobutamine, norepinephrine, IVIG, aspirin, prednisolone	Recovery
Bastug, 2021, Turkey [24]	40, male, Caucasian	None	Fever (39 °C), tachycardia, tachypnea, abdominal pain, diarrhea, and skin rash	23 days prior	PCR (−) IgM (+) IgG (+)	NR	Lymphopenia, leukocytosis as well as high liver function tests, ferritin, d-dimer, troponin, BNP, CRP, fibrinogen, PCL, and IL-6	CT abdomen: inflamed intestine and mesentery, mesenteric lymphadenopathy, and effusion TTE: global hypokinesia, reduced left ventricle function (EF 45%), and mild pericardial effusion. EF increased to 60% and the effusion resolved after treatment	Antibiotics, methylprednisolone, IVIG, full-dose enoxaparin	Recovery
Sokolovsky, 2021, [31]	36, female, Hispanic	None	Fever, vomiting, abdominal pain, diarrhea, arthralgia, rash hypotension, and tachycardia	No	PCR (+) Abs(+)	NR	Elevated liver enzymes, direct bilirubin, albumin, CRP, ferritin, d-dimer, ESR, and hyponatremia (115 mmol/L)	TTE: normal EF (65%) and moderate tricuspid regurgitation CTA coronaries: normal with rim pericardial effusion CT chest: trace pleural effusion	Steroids, acetylcysteine, IVIG, aspirin	Recovery
Julius, 2021, [11]	59, female, Caucasian	HTN and dyslipidemia	Fever, right cervical lymph node swelling, odynophagia, hypotension, and rash (neck and chest)	20 days prior, PCR (+)	PCR (+)	Yes	Slightly elevated AST, ALT; high Tn, CRP, and ferritin	CT neck: enlarged right nodes with one exhibiting liquefaction EKG: ST elevation in V1 and V2	Antibiotics, steroids, norepinephrine, epinephrine, terlipressin mechanical ventilation	Deceased
Parpas, 2021 [32]	67, male	HTN, cirrhosis	Dyspnea weakness, weight loss, anorexia, nausea, extremities edema, tachycardia, and cognitive impairment	68 days prior	PCR (−) Abs (+)	NR	Low sodium (109 mEq/L) and albumin (3 g/dL), leukocytosis (35,000/mm^3^). High d-dimer, LDH, and PCL	Chest X-ray: bilateral basal infiltrative lesions CT chest: lung atelectasis/collapse TTE: Pulmonary HTN, and grade I diastolic dysfunction Duplex of lower limbs: no DVT Renal biopsy: moderate to severe acute tubular necrosis	Antibiotics, unfractionated heparin, dexamethasone, and hemodialysis	Recovery
Pérez, 2021, [25]	88, male	HTN, dyslipidemia, essential tremors	Hypoxia (saturation 87%), dyspnea, and peripheral edema	54 days prior PCR (+) Abs (+)	PCR (−) IgM (+) IgG (+)	NR	Creatinine (2.14 mg/dL), proteinuria (> 600 mg/dL), and low albumin 3 g/dL High LDL, CRP, and d-dimer	Chest X-ray: typical COVID-19 picture and pleural effusion Renal biopsy: findings suggesting acute IgA-dominant infection-associated glomerulonephritis	Intravenous furosemide, intravenous methylprednisolone	Recovery
Balan, 2021, [33]	46, male	Obesity (BMI 42 kg/m^2^)	Hypotension, hypoxia tachypnea, right hemiparesis, ataxia, and left hemianesthesia	60 days prior	PCR (−) Abs (+)	Yes	Elevated ferritin, CRP, LDH, PCT, high creatinine (4.1 mg/dL) and Tn	TTE: normal EF and elevated right ventricular pressures CT chest: bilateral apical and basal as well as right middle ground-glass opacities	Norepinephrine, antibiotics unfractionated heparin, dexamethasone, tocilizumab, hemodialysis	Deceased
Mieczkowska, 2021, [22]	32, male	None	Fever, tachycardia, right-sided swollen groin lymph nodes, diarrhea, and palms and soles rash	Two months prior	PCR (−) IgG (+)	No	Elevated AST, ALT, and direct bilirubin. Elevated inflammatory markers (CRP, ferritin, PCL, IL-6, ESR, and d-dimer)	TTE: EF 55% and pericardial effusion CT: lymphadenopathy of the right groin	Enoxaparin and intravenous methylprednisolone	Recovery
Mieczkowska, 2021, [22]	43, female	None	Fever, myalgia, headache, cough, and skin rash. Hypotension, cardiomyopathy, and acute kidney injury	No	PCR (−) Serology (+)	NR	Leukocytosis (21,500/mm^3^). Elevated ESR, CRP, ferritin, and d-dimer. Elevated AST, ALT, and ALP	Chest X-ray: right basal pneumonia Abdominal ultrasound: pericholecystic fluid, hepatomegaly, and steatosis TTE: EF 40%	Vasopressors, antibiotics, intravenous heparin, methylprednisolone	Recovery
Hékimian, 2021 [12]	40, male	DM (BMI 26 kg/m^2^)	Apyretic, dyspnea, severe asthenia	No	PCR (+) IgG (−)	Yes	Elevated PCT, CRP, ferritin Elevated AST, ALT, and ALP Elevated LDH, CPK Peak troponin 439 ng/L Peak BNP 6025 pg/mL	Chest CT: severe multifocal PNA TTE: EF 45%	Mechanical ventilation, dobutamine, norepinephrine, ECMO	Recovery
Hékimian, 2021 [12]	19, female	None (BMI 24 kg/m^2^)	Fever, dyspnea, cough	No	PCR (−) IgG (+)	Yes	Elevated CRP, ferritin, LDH Peak troponin 10,652 ng/L Peak BNP 2585 pg/mL	Chest CT: mild infiltrates TTE: EF 30%	Mechanical ventilation, dobutamine, norepinephrine, ECMO	Recovery
Hékimian, 2021 [12]	22, male	DM, asthma (BMI 38 kg/m^2^)	Fever, dyspnea, cough, severe asthenia	No	PCR (−) IgG (−)	Yes	Elevated CRP, ferritin, LDH Peak troponin 166 ng/L	Chest CT: severe infiltrates TTE: EF 30%	Mechanical ventilation, ECMO	Recovery
Hékimian, 2021 [12]	19, male	None (BMI 22 kg/m^2^)	Fever, headache, diarrhea, dyspnea, severe asthenia	No	PCR (−) IgG (+)	Yes	Elevated CRP, ferritin, LDH Peak troponin 806 ng/L Peak BNP 26,956 pg/mL	Chest CT: negative TTE: EF 15%	Dobutamine, norepinephrine	Recovery
Hékimian, 2021 [12]	16, male	None (BMI 18 kg/m^2^)	Fever, anosmia, abdominal pain, rash to hands and feet, conjunctivitis, strawberry tongue, adenopathy, severe asthenia, chest pain	No	PCR (+) IgG (+)	Yes	Elevated CRP, ferritin, LDH Peak Troponin 2545n ng/L	Chest CT: mild infiltrates TTE: EF 20%	Mechanical ventilation, dobutamine, norepinephrine, IVIG	Recovery
Hékimian, 2021 [12]	16, female	None (BMI 24 kg/m^2^)	Fever, headache, abdominal pain, rash to hands and feet, dyspnea, severe asthenia	Yes, anosmia and cough 1 month prior	PCR (−) IgG (+)	Yes	Elevate CRP, ferritin, and LDH Peak troponin 64 ng/L Peak BNP 1689 pg/mL	Chest CT: negative TTE: EF 45%	None	Recovery
Hékimian, 2021 [12]	17, male	Moderate aortic regurgitation (BMI 32 kg/m^2^)	Fever, headache, abdominal pain, diarrhea, dyspnea, severe asthenia, conjunctivitis	No	PCR (+) IgG (+)	Yes	Elevated ferritin and LDH Peak troponin 138 ng/L Peak BNP 35,000 pg/mL	Chest CT: mild pulmonary edema TTE: EF 20%	Mechanical ventilation, dobutamine, norepinephrine, IVIG, corticosteroids 2 mg/kg/day	Recovery
Hékimian, 2021 [12]	25, female	None (BMI 23 kg/m^2^)	Fever, headache, abdominal pain, dyspnea, severe asthenia, myalgias, arthralgias, adenopathy	No	PCR (−) IgG (+)	Yes	Elevated CRP, ferritin, LDH Peak troponin 2542 ng/L Peak BNP 24,540 pg/mL	Chest CT: negative TTE: EF 50%	Nasal cannula	Recovery
Hékimian, 2021 [12]	17, female	None (BMI 18 kg/m^2^)	Chest pain, dyspnea	No	PCR (+) IgG (+)	Yes	Elevated CRP, ferritin, LDH Peak troponin 4905 ng/L Peak BNP 3362 pg/mL	Chest CT: pulmonary edema TTE: 20%	Mechanical ventilation, dobutamine, norepinephrine, ECMO, IVIG, corticosteroids 2 mg/kg/day	Deceased
Hékimian, 2021 [12]	37, male	HTN (BMI 35 kg/m^2)^	Fever, headache, diarrhea, severe asthenia	No	PCR (−) IgG (+)	Yes	Elevated ferritin, LDH Peak troponin 1164 ng/L Peak BNP 35,000 pg/mL	Chest CT: Negative TTE: EF 45%	IVIG, corticosteroids 2 mg/kg/day	Recovery
Hékimian, 2021 [12]	29, female	None (BMI 22 kg/m^2^)	Fever, abdominal pain, diarrhea, rash, conjunctivitis, severe asthenia	Yes, 1 month earlier	PCR (−) IgG (+)	Yes	Elevated CRP, ferritin, LDH Peak troponin 200 ng/L Peak BNP 21,298 pg/mL	Chest CT: negative TEE: EF 50%	IVIG	Recovery

*PMH* past medical history, *HTN* hypertension, *BMI* body mass index, *BPM* beats per minute, *MIS-A* multisystem inflammatory syndrome in adults, *PCT* procalcitonin, *AST* aspartate transaminase, *ALT* alanine transaminase, *ALP* alkaline phosphatase, *CRP* C-reactive protein, *ESR* erythrocyte sedimentation rate, *LDH* lactate dehydrogenase, *EKG* electrocardiogram, *CAP* community-acquired pneumonia, *PNA* pneumonia, *HD* hospital day, *ANA* antinuclear antibodies, *ANCA* antineutrophil cytoplasmic antibodies, *OD* once daily, *Tn* troponin, *BNP* brain natriuretic peptide, *DVT* deep vein thrombosis, *TTE* transthoracic echocardiogram, *EF* ejection fraction, *MRI* magnetic resonance imaging, *MV* mechanical ventilation, *CRRT* continuous renal replacement therapy, *IVIG* intravenous immunoglobulins, *LMWH* low molecular weight heparin, *Abs* antibodies, *SatO*_*2*_ saturation of O_2_

Cardiovascular impairment was also noted in the literature. Specifically, tachycardia (22/36; 61%) and hypotension/cardiogenic shock with documented impaired ejection fraction (23/36; 64%) [5–8, 10, 12, 15, 17–24]. The left ventricular function/ejection fraction normalized with treatment in 15 patients [6, 7, 12, 17, 21, 23, 24], of whom 7 patients received IVIG with or without aspirin [10, 12, 17, 23, 24]. Overall, 28/36 (78%) patients recovered and were safely discharged. Cardiac MRI has been discussed in the literature in terms of assessing for myocarditis. It can confirm signs of diffuse myocardial inflammation while ruling out ischemic or stress-induced cardiomyopathy [12].

There is no consensus on the mechanism causing MIS-A during or post-CoVID-19 infection. MIS-A is viewed as an atypical immune response causing systemic vasculitis and multiple acute organ injury. The dramatic response to IVIG and high-dose aspirin supports the occurrence of vasculitis, which was demonstrated in our patient. She was successfully weaned off vasopressors following the IVIG treatment, and discharged without any complications in her hospital course. Target management of MIS-A with immunomodulatory therapy has reversed acute kidney injury [25] and heart failure, with normalization of cardiac function in many patients [6, 7, 12, 17, 21, 23, 24]. Many theories were proposed to uncover the linkage between vasculitis and SARS-CoV-2 infection. For example, IL-6 increases markedly during CoVID-19 infection, and it is the same cytokine that mediates vasculitis in Kawasaki syndrome. IL-6 enhances the adhesion of lymphocytes to endothelial cells causing their damage [26]. Another theory points toward complement activation and capillary deposition of immune complexes as initial insult, which could be suggested in our case based on her low complement C3 and C4 levels [27].

MIS-A of CoVID-19 shares many similarities with Kawasaki-like multisystem inflammatory syndrome, a syndrome which has been linked to other viral infections. Diagnosis of Kawasaki disease requires (1) fever for ˃ 5 days and (2) at least four signs of conjunctivitis, involvement of the oropharyngeal mucosa or IgA infiltration of the upper respiratory tract, cervical lymphadenopathy, rash, and extremity changes (edema or erythema) [28]. Furthermore, Kawasaki may present with acute kidney injury or aneurysms, especially in coronaries and abdominal aorta.

COVID-19 Kawasaki-like syndrome is diagnosed by (1) fever for ˃ 3 days, (2) at least two signs of rash, hypotension/shock, or acute cardiac injury (infarction, pericarditis, left ventricle dysfunction, right ventricular dysfunction, or coronary syndrome), (3) coagulopathy, or (4) acute gastrointestinal (GI) symptoms in the setting of elevated inflammatory markers (CRP, d-dimer, and/or ferritin) during or after COVID-19 infection, after excluding other infections [29]. This description was consistently seen with our patient. She exhibited fever, strawberry-like rash, hypotension requiring vasopressors, decreased ejection fraction, nephropathy, and significant elevations in her CRP and d-dimer.

**Fig. 1:**
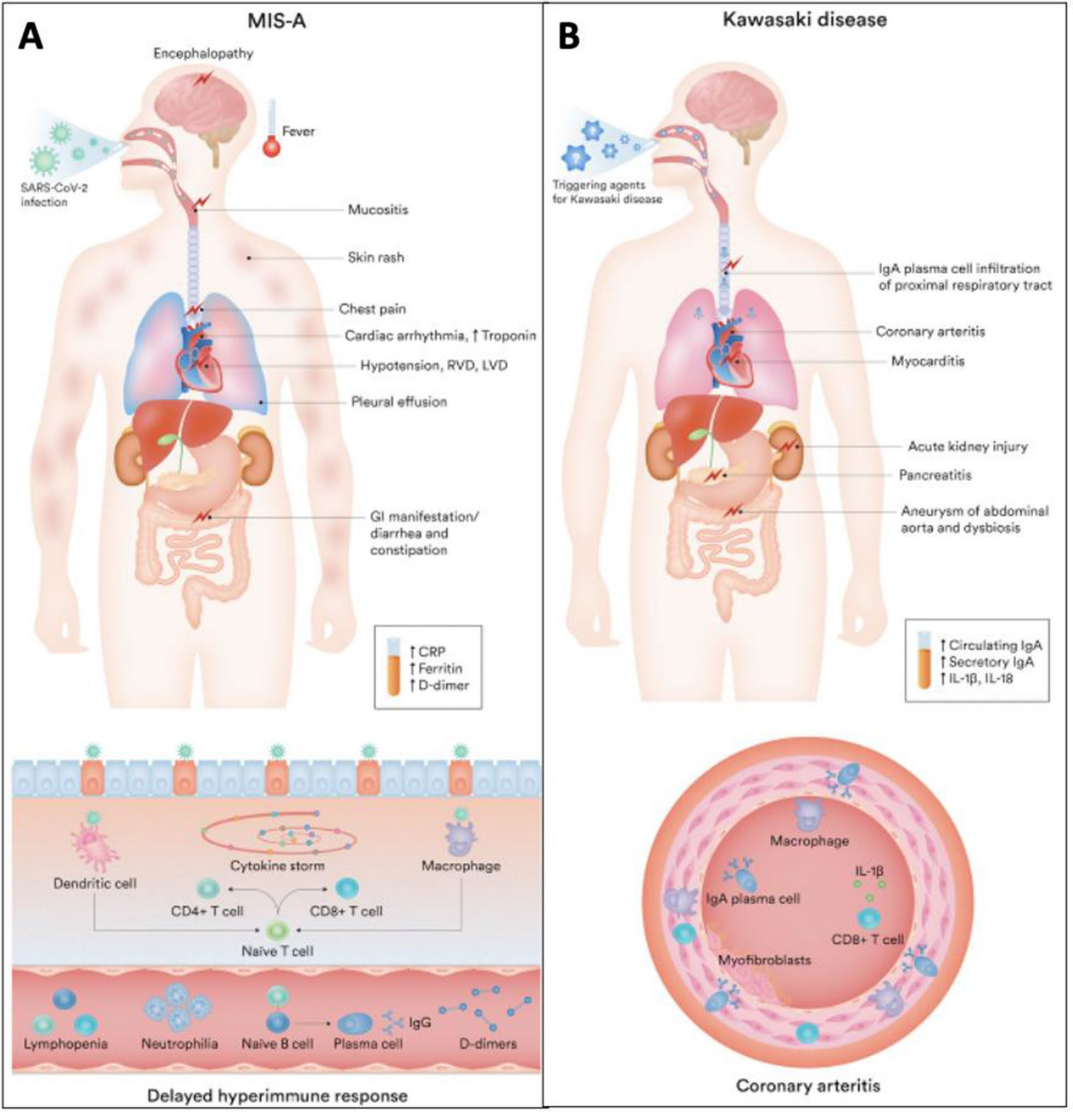
Clinical manifestations and possible mechanism of injury in COVID MIS-A and Kawasaki disease. **A** MIS-A. **B** Kawasaki Disease. *MIS-A* multisystem inflammatory syndrome in adults, *RVD* right ventricular dysfunction, *LVD* left ventricular dysfunction, *GI* gastrointestinal, *CRP* C-reactive protein, *IgG* immunoglobulin G, *IgA* immunoglobulin A, *IL* interleukin. This figure was created by Fardad Behzadi for the purposes of this publication

Figure 1 illustrates the clinical features and possible pathophysiology basis of MIS-A and classic Kawasaki syndromes. Our patient did not fulfill the criteria of classic Kawasaki. Furthermore, the acute cardiac injury and hypotension, acute renal injury, fever, sore throat, unilateral lymphadenopathy, and elevated inflammatory markers in the setting of positive SARS-CoV-2 IgG antibody support a diagnosis of MIS-A.

In terms of management, there was considerable variation in treatment modalities when reviewing the literature. In our case, the patient was aggressively fluid resuscitated and started on broad spectrum antibiotics, steroids, and ultimately vasopressors. In conjunction with the infectious disease team, full-dose aspirin and IVIG was initiated, with resolution of her symptoms and ultimate discharge. To demonstrate the variability in treatments, we reviewed previously documented cases of MIS-A. Summarizing Table 2, 44% of patients were given IVIG, 56% given steroids, 39% antibiotics, 13% given immunomodulators (tocilizumab, anakinra, cyclophosphamide, rituximab), 11% given aspirin, 22% anticoagulation, and 36% requiring vasopressors. Despite the differences in management, recent literature studying the treatment modalities of MIS-C concluded that were was no evidence that IVIG alone or IVIG with steroids or immunomodulators leads to higher rates of recovery [30]. These findings may not be generalizable to the adult population who experience MIS-A, but it gives insight into the challenges of choosing a treatment modality.

## Conclusion

Our case report is an example of the presentation, diagnosis, and management of MIS-A. As we dove into the literature and discovered other documented cases of MIS-A, we created Fig. 1 to illustrate the similarities and differences when compared with Kawasaki-like multisystem inflammatory syndrome. Our research into previous case reports illustrates the wide range of presentations, degree of end-organ damage, and treatment modalities. This diagnosis needs to be considered in the presence of recent COVID infection with new onset end organ failure, as prompt diagnosis and treatment is crucial for better outcomes.

## Data Availability

Not applicable.

## Abbreviations

MIS-A: Multisystem inflammatory syndrome in adults
ED: Emergency department
ICU: Intensive care unit
IVIG: Intravenous immunoglobulin
BMI: Body mass index
PCR: Polymerase chain reaction
CT: Computed tomography
POCUS: Point of care ultrasound
CRP: C-reactive protein
AST: Aspartate aminotransferase
ALT: Alanine aminotransferase
ALP: Alkaline phosphatase
BNP: Brain natriuretic peptide
CDC: Centers for Disease Control
ESR: Erythrocyte sedimentation rate
IL-6: Interleukin-6
